# Magnetization Lifetimes Prediction and Measurements Using Long-Lived Spin States in Endogenous Molecules

**DOI:** 10.3390/molecules25235495

**Published:** 2020-11-24

**Authors:** F. Teleanu, C. Tuță, A. Cucoanes, S. Vasilca, P. R. Vasos

**Affiliations:** 1 Extreme Light Infrastructure—Nuclear Physics ELI-NP, “Horia Hulubei” National Institute for Physics and Nuclear Engineering IFIN-HH, 30 Reactorului Street, 077125 Bucharest-Magurele, Romania; florin.teleanu@eli-np.ro (F.T.); andi.cucoanes@eli-np.ro (A.C.); 2 Interdisciplinary School of Doctoral Studies (ISDS), University of Bucharest, Mihai Kogalniceanu Street 36-46, 050067 Bucharest, Romania; 3 “Horia Hulubei” National Institute for Physics and Nuclear Engineering IFIN-HH, 30 Reactorului Street, 077125 Bucharest-Magurele, Romania; catalin.tuta@nipne.ro; 4 College for Advanced Performance Studies and Faculty of Chemistry and Chemical Engineering, Babes-Bolyai University, Mihai Kogalniceanu Street 1, 400028 Cluj-Napoca, Romania; 5 Department of Analytical Chemistry Faculty of Chemistry, University of Bucharest, 90-92 Panduri Ave., 050067 Bucharest, Romania

**Keywords:** biomarkers, NMR, long-lived states, amino acids

## Abstract

Nuclear magnetization storage in biologically-relevant molecules opens new possibilities for the investigation of metabolic pathways, provided the lifetimes of magnetization are sufficiently long. Dissolution-dynamic nuclear polarization-based spin-order enhancement, sustained by long-lived states can measure the ratios between concentrations of endogenous molecules on a cellular pathway. These ratios can be used as meters of enzyme function. Biological states featuring intracellular amino-acid concentrations that are depleted or replenished in the course of in-cell or *in-vivo* tests of drugs or radiation treatments can be revealed. Progressing from already-established long-lived states, we investigated related spin order in the case of amino acids and other metabolites featuring networks of coupled spins counting up to eight nuclei. We detail a new integrated theoretical approach between quantum chemistry simulations, chemical shifts, *J*-couplings information from databanks, and spin dynamics calculations to deduce *a priori* magnetization lifetimes in biomarkers. The lifetimes of long-lived states for several amino acids were also measured experimentally in order to ascertain the approach. Experimental values were in fair agreement with the computed ones and prior data in the literature.

## 1. Introduction

Predictive modeling of cancer cell sensitivity to various therapies can be based on metabolomics [1]. Equilibria of concentrations of endogenous small molecules can be both the motor and most sensitive diagnostic of biological transformations. The responses of the central nervous system and different organs to amino-acid depletion regimens [2] are actively studied with both molecular- and physiological-scale investigation tools. Transient ratios of endogenous molecule concentrations are the new frontier of in vivo real-time enzymology in tests of drug and radiation therapy [3]. Accessible via new experimental procedures such as non-invasive dissolution-dynamic nuclear polarization (*d*-DNP) for functional metabolic imaging [4]. Advances in hyperpolarization via *d*-DNP [5] allow us to generate magnetization up to 10,000 times larger than conventional Magnetic Resonance (MR). Such enhanced magnetization, once generated, needs to be preserved. In this context, new collective spin states with long lifetimes, constructed based on molecular symmetry, can render *d*-DNP-MR suitable for imaging molecular transformations in vivo [6,7,8,9,10,11,12]. The advancement of nuclear spin hyperpolarization techniques has opened new paths for studying enzyme activity in cell [10,11] by drastically increasing the signal intensities for relevant molecules across metabolic pathways. Nuclear hyperpolarization methods are strongly limited by the lifetime of spin configurations, which is of the order of seconds in most molecules. Therefore, new solutions for magnetization storage are desirable, especially for biological molecules. Transverse magnetization obtained via standard NMR excitation—a 90 degrees pulse—is, in most molecules, rapidly lost due to spin–spin relaxation. Magnetization avatars other than those based on transverse forms can feature increased lifespans. Longitudinal magnetization is one such form, as its decay time constant, *T*_1_, is longer than that of transverse magnetization (*T*_2_) (i.e., *T*_1_ > *T*_2_), even though for small molecules in currently-achieved homogeneous magnetic fields, these two time constants are very similar [12]. Carefully-designed states can display long lifetimes of spin order, just like the now classic long-lived states (LLS) in pairs of coupled spins, which can feature relaxation time constants more than forty times longer than their spin-lattice relaxation time constant, *T*_LLS_ > 40*T_1_ [13,14]. A strong motivation for exploring long-lived spin configurations in metabolites is that magnetization lifetimes can be used as filters to separate compounds of interest in the crowded cell background [15]. Long-lived states on selected biomolecules can afford an image contrast proportional to the ratio between their lifetimes and longitudinal relaxation times in other cell compounds, *T*_LLS_(biomolecule)/*T*_1_(background).

The simplest form of LLS is a two-spin based difference between the population of the singlet state (SS) and the average of the populations of the triplet states [16,17,18,19,20]. SS and triplet states become eigenfunctions of the Hamiltonian describing the magnetic interactions of two *J*-coupled spin-½ nuclei, I and S, in a molecule in the liquid phase, provided that the scalar coupling between I and S, $J_{IS}$, far exceed the difference in frequency between the two spins,$\;J_{IS}>>\Delta \upsilon_{IS}$. The field dependence of the resonance frequency indicates that, in order to render the singlet and triplet states eigenfunctions of the nuclear spin Hamiltonian, either a vanishingly small main magnetic field *B*_0_ is needed, or the two spins must be rendered magnetically equivalent by applying a radio-frequency field with an amplitude sufficiently large to mask their chemical shift difference, $\upsilon_{1}>>\Delta \upsilon_{IS}$. The three triplet eigenfunctions of the two-spin system are symmetric with respect to the permutation between the two spins. The SS is antisymmetric with respect to this permutation, which makes it magnetically inactive in a first-order approximation.

Molecules adopt singlet and triplet states for their nuclear magnetic wavefunction according to a probability given by the Boltzmann distribution, but these molecular spin ensemble populations can be modified during the NMR experiment. Once the singlet/triplet population equilibrium is perturbed, the states decay back to this equilibrium via ‘phosphorescent’ communication between singlet and triplet states through relaxation or coherent evolution given by the difference in chemical shift between the two nuclei. In the former eigenbasis, the phosphorescent singlet–triplet transition is particularly slow because the strongest relaxation mechanism, dipole–dipole interaction, is spin-permutation symmetric and therefore cannot convert an antisymmetric state into a symmetric state [12]. The “leaked” spin population via singlet–triplet transitions is observable once the system is rendered inequivalent ($\Delta \upsilon_{IS}$ ≠ 0) from the point of view of the (I,S) pair. Key to LLS experiments is the ability to switch between the two eigensystems, either switching on and off the main field *B*_0_ or the radio-frequency sustaining field of amplitude, $\upsilon_{1}$. In our experiments, we opted for the latter approach. This flexibility of LLS experiments renders possible in-cell, in-vivo and clinical imaging with molecules that are prepared in magnetic ensembles featuring large singlet–triplet population differences (i.e., hyperpolarized) [5,17,18].

In the Liouville space associated to a pair of isolated, *J*-coupled spins (I,S), the normalized singlet–triplet population difference, known as a long-lived state, *Q*_LLS_, is the eigenstate whose eigenvalue has the lowest real component except for the identity operator and no imaginary component. When external couplings arise for the I or S spin, the singlet state loses, in part or entirely, depending on the strength of the external perturbation [19], its eigenfunction character and its magnetization storage capability. Within extended networks of coupled spins, relaxation-favored states resembling *Q*_LLS_ can be identified. An extended system of coupled spins where any of the spins has at least one *J* coupling within the network may also possess other eigenstates with prolonged lifetime. Long-lived states in systems comprising more than two coupled spins [20] correspond to eigenstates of the Liouvillian that are stationary during coherent evolution sustained by a radio-frequency field as they commute with the scalar-coupling Hamiltonian. These states are challenging to access via spin dynamics due to their complex expression in terms of angular spin momentum.

In this manuscript, we explore the lifetime of long-lived states in simple endogenous molecules that are suitable for in cells or in vivo NMR experiments acting as biomarkers of metabolic processes using theoretical and experimental approaches. This analysis provides, via computational techniques, maximal relaxation time constant values attainable in spin networks that are more complex than (I,S) pairs. The methodology was benchmarked comparing predicted longitudinal relaxation time constants with experimentally-determined ones. The lifetimes of long-lived states were determined for several amino acids and the results compared with both computational predictions and previous work [20,21,22,23,24,25,26,27].

## 2. Results

**Figure 1:**
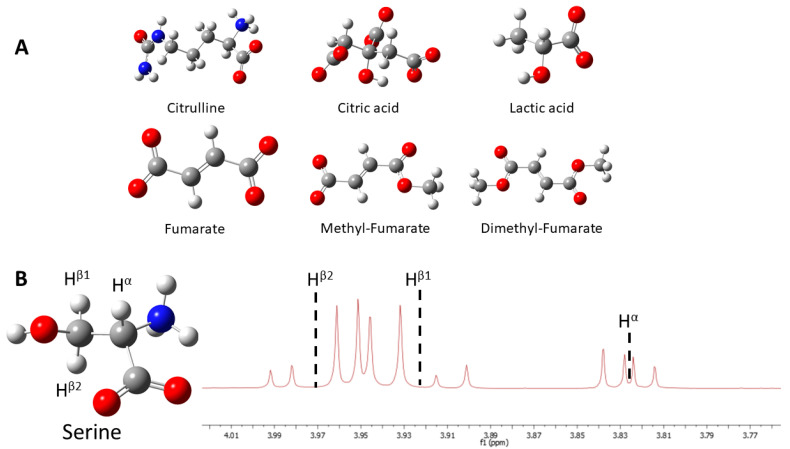
(**A**) The structures of theoretically studied endogenous molecules. (**B**) The structure of serine with its three-spin system and proton spectrum.

Our investigations first addressed several amino acids from both experimental and theoretical perspectives, in order to gain insights into the applicability of long-lived states on coupled proton networks inside their structure, where such networks reached up to eight spins (in phenylalanine). Other endogenous molecules relevant for metabolomics were examined, such as fumarate and citric acid [16,28] (see Figure 1).

Our approach consisted of predicting two sets of data for the selected biomolecules: (i) computer generation of the slowest-relaxing state that exists in the molecule according to Liouville-space diagonalization (though this exact state may not exist as such in reality) and (ii) generation of bilinear combinations of product operators that are known to feature long-lived states and are easy to attain experimentally. The Liouville-space diagonalization was also used to predict spin-lattice relaxation time constants for most biomolecules (all the amino acids for which results are reported in Table 1) for benchmarking purposes. Theoretical predictions for LLS values were also compared with experiments for several molecules (Ser, Cys, Asn). All values reported in Table 2 contain predictions for relaxation rates using the same computational approach that was benchmarked in Table 1.

The long-lived states that were identified by our theoretical approach in selected molecules (Table 1) corresponded to the second smallest eigenvalue of the Liouvillian for each nuclear system (the smallest one corresponds to the identity operator, which is not relevant). To highlight the slow relaxation rate of the long-lived states in the case of amino acids, we evaluated the longitudinal relaxation time constants (*T*_1_) for the H^β^ protons with respect to the carboxylic group for comparison. In Table 1, we list the relaxation times for each studied amino acid denoted with its usual abbreviation. Computed magnetization lifetimes were in fair agreement with the experiments for the case of *T*_1’_s. The results for the β protons indicate that the simulation method is suitable for good approximation of the long-lived states’ lifetimes.

All long-lived states displayed a considerable lifetime enhancement *T*_LLS_/*T*_1_ for the aliphatic protons (calculated here at the β position). Our calculations suggest that a decrease in the external magnetic field changes the nature of LLS in some cases (Asp, Asn, Cys, Ser) as well as increases their lifetimes without any considerable effects for the other molecules.

**Figure 2:**
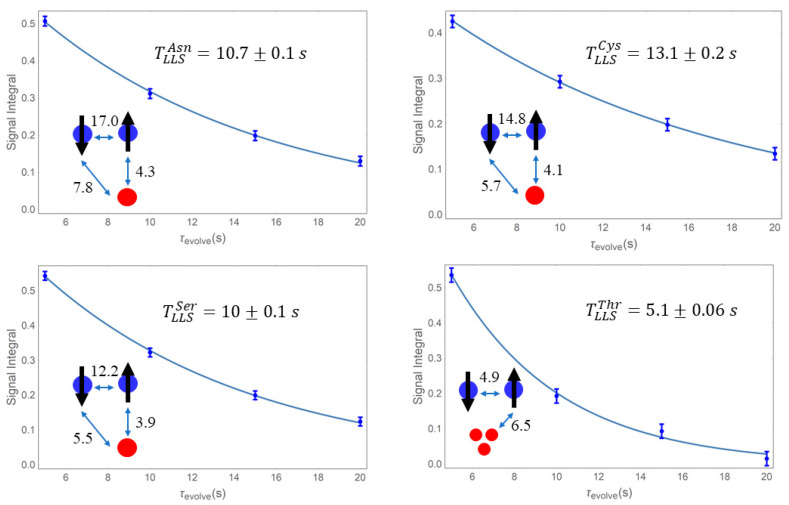
Time evolution of long-lived states excited using the pulse sequence described in the Methods section (Figure 3) and sustained with continuous-wave radiation in asparagine, cysteine, serine, and threonine for periods of $\tau_{evolve}\in \left\{5\;s,\;10\;s,\;15\;s,\;20\;s\right\}$. Determined lifetimes, *T*_LLS_, were derived from a Monte Carlo fitting of an exponential function, $I\left(t\right)=I\left(0\right)*\exp\left(-\frac{\tau_{evolve}}{T_{\mathrm{LLS}}}\right)$; Error bars are proportional to the noise of each spectrum; for each molecule, spin systems are shown along with the known values for scalar coupling constants within the system (adjacent to arrows, in Hz).

**Figure 3:**
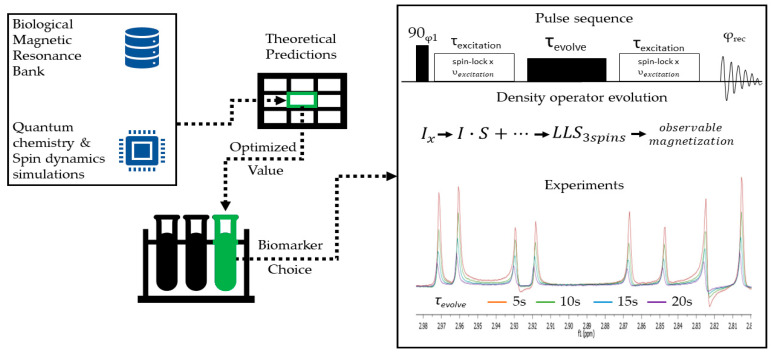
Schematic representation of the methodology developed for choosing molecules suited to sustain long-lived states with lifetimes comparable to those of metabolic conversions in vivo. First, the investigated molecules were evaluated from a theoretical point of view by simulating the corresponding spin systems. Magnetic parameters were extracted from data banks and atomic coordinates from quantum-chemistry simulations. Then, the system’s Liouvillian was computed and diagonalized. The eigenstate with the smallest non-zero eigenvalue was identified as the system’s longest-lived state. The best-suited molecules were then chosen for experiments and the a priori computed lifetimes were compared to experimental lifetimes measured using an NMR spectrometer.

An experimental measurement of the lifetimes of long-lived states was conducted for the case of asparagine, cysteine, serine and threonine (Figure 2) using the spin-lock induced crossover (SLIC) method [28,29]. The pulse sequence for this method is given in Figure 3. The selected parameters were as follows: the carrier offset was set to the average value of the H^β^ chemical shifts; the amplitude of the spin-lock pulse was set equal to the scalar coupling between the two H^β^ protons; the duration of the spin-lock was chosen according to the original SLIC paper [28] as $\tau_{excitation}=0.707/\Delta \upsilon$, where Δυ is the difference between the H^β^ frequencies. Between the two spin-lock periods, the two H^β^ are rendered magnetically equivalent by a continuous wave irradiation at an amplitude equal to 5Δυ [29]. Five different delays for the sustaining period were chosen $\tau_{evolve}\in \left\{5\;s,10\;s,15\;s,20\;s\right\}$. The superimposed spectra are given in Figure 3. The experiments exciting spin distributions using this straightforward approach that approximates 3-spin systems as their counterparts on the two spins featuring the largest *J*-coupling may, in extreme cases where one of the *J*-couplings in the triad largely overweighs the other two, give rise to a singlet order between those two nuclei, which is slightly perturbed by the third spin [19,20]. There was good agreement between the computed value of $T_{LLS}^{Ser}=9.47s$ and the experimental one determined by this method $T_{LLS}^{Ser}\approx 10\;s$ (Figure 2).

Through a similar approach, the lifetimes of LLS for the case of Asn, Cys, and Thr were determined experimentally as $T_{LLS}^{Asn}\approx 10.7\;s$, $T_{LLS}^{Cys}\approx 13.1\;s$, $T_{LLS}^{Thr}\approx 5.1\;s$, respectively, in fair agreement with the computed ones (Figure 2).

For the case of metabolic-relevant molecules other than the amino acids used for benchmarking, the same methodology was employed. All aliphatic protons were considered for the creation of the Liouvillian. The LLS is identified as the eigenstate with the second smallest eigenvalue. For some specific cases (i.e., fumarate, dimethylfumarate), the exact LLS is simply given by a singlet–triplet population imbalance, as there exists an isolated pair of *J*-coupled protons. These LLS are immune to dipolar relaxation mechanisms between the coupled *J*-coupled spins so their lifetime is much longer than *T*_1_ computed for the same protons.

From Table 1, one can easily see that fumaric acid and its derivatives are promising candidates for diffusion studies or metabolomics, given that fumaric acid enters metabolic transformations in the Krebs cycle. The predicted long lifetime should only be considered as indicative because, in our simulations, only the intramolecular dipolar relaxation was considered with no interferences from other surrounding molecules. In such real-life scenarios where molecules bump into each other, the intermolecular dipolar coupling would relax the LLS in fumarate and dimethylfumarate and their lifetime would be long, yet finite. The main issue with using fumarate or dimethylfumarate is the chemical equivalency of the two aliphatic protons, which makes the excitation of LLS impossible. This can be circumvented by rendering them chemically different with the help of chemical reaction (i.e., addition of HX molecule with X = OH, Cl). This type of approach can combine the long-lived propensity of magnetization in such biomarkers with the enhanced signal given by DNP techniques. On the other hand, one can exploit the difference in chemical shielding of the two central protons in the case of monomethylfumarate and excite a long-lived state that will feature a moderate lifetime.

Furthermore, we investigated long-lived states with bilinear combinations of 3-spin operators in a manner similar to prior studies [20]. As shown by Equation (1), there is a specific choice of product operator coefficients that maximizes the relaxation time of $Q^{ISR}$ states. These states are true eigenstates of a three-spin system only when the evolution under Zeeman interaction is suppressed, that is, the sample is taken out of the magnet. For this purpose, we chose the H^β^ nuclei (I and S) and H_α_ nucleus (R) in those molecules where all three were isolated from other non-exchangeable protons and the external magnet turned to zero. Among the eigenstates of such spin systems, we chose the most similar to that of Equation (1) and we projected the product spin operator $\vec{I}\cdot \vec{S}$, $\vec{I}\cdot \vec{R}$ and $\vec{S}\cdot \vec{R}$ to obtain the λ coefficients (see Table 2).

The largest contribution for these bilinear states can be attributed to the spin-product operator between the strongest-coupled nuclei ($\vec{I}\cdot \vec{S}$). This is to be expected as the contribution from the third spin can be regarded as a residual interaction to a pure singlet state given the fact that the other coupling constants *J_IR_* and *J_SR_* are much smaller than the *J_IS_.* These *Q^ISR^* states displayed improved lifetimes in low magnetic field $B_{0}=1\;T$, which makes them good candidates for studying biochemical transformations in vivo.

## 3. Materials and Methods

*T*_1_ and *T*_LLS_ measurements were performed by inversion-recovery experiments and respectively, a method based on spin-lock induced crossover (SLIC) [28] for LLS excitation (Figure 3) for amino acids dissolved in 100% deuterated water at T = 304 K on a Bruker Avance spectrometer operating at *B*_0_ = 9.36 T (Larmor frequency $\upsilon_{H}=400\;\mathrm{MHz}$), equipped with a 5-mm BBO BB/19F/^1^H/D probe. Chemical shifts and *J*-couplings were retrieved from the Biological Magnetic Resonance Bank [30]. The efficiency of SLIC-based methods was compared for benchmarking purposes with hard pulses and gradients [20,31]. For the SLIC experiments, the pulse parameters must be adapted to each molecule’s specific scalar couplings and chemical shifts. The amplitudes for the excitation pulse were equal to the maximum value of the scalar coupling in the spin network, $\upsilon_{excitation}=Max\left(J_{i,j}\right)$, with a pulse duration $\tau_{excitation}=\frac{0.707}{\Delta \upsilon_{IS}}$, where $\Delta \upsilon_{IS}$ is the chemical shift difference between the strongest coupled spins ($J_{IS}=Max\left(J_{i,j}\right)),$ which are further denoted as I, respectively S. The carrier was set to the average of the two chemical shifts $\upsilon_{av}=\frac{\upsilon_{I}+\upsilon_{S}}{2}$. The amplitude of the sustaining field was $\upsilon_{1}=5\Delta \upsilon_{IS}$, which proved to be sufficient in other studies [29]. Sustaining delays $\tau_{evolve}\in \left\{5s,10s,15s,20s\right\}$ were used to measure the lifetime of LLS. The relevant resulting signal intensities were integrated, and the integrals fitted with an exponential function $I\left(t\right)=I\left(0\right)*\exp\left(-\frac{t}{T_{\mathrm{LLS}}}\right)$. The phase-cycling for the first 90° pulse $\varphi_{1}$ and the detection $\varphi_{rec}$ is as follows: $\varphi_{1}=\left(y,-y\right)$ and $\varphi_{rec}=\left(y,-y\right)$.

Several amino acids and other endogenous molecules suited for studying metabolic pathways were investigated. In order to compute the relaxation properties of the nuclear spin states in these molecules, the magnetic parameters (chemical shifts and *J*-couplings in the system) and the spatial distribution of the atoms are needed. The most significant relaxation mechanism for the case of proton systems are dipolar interactions, which act through space. In order to find the inter-atomic distances between atoms, molecular structures were optimized using quantum chemistry calculations software Gaussian 09 [32] using the density functional theory (DFT) with the Perdew–Burke-Ernzerhof (PBE0) hybrid functional along with the triple-ζ basis set with polarization (‘def2tzvp’). In order to ensure that we obtained an energetic minimum for every molecule, a frequency analysis was performed: all presented structures lacked imaginary frequencies, hence they represent geometries with minimum energies. The integration grid ‘UltraFine’ was chosen in order to provide accurate and consistent numerical calculations.

The geometries of the aliphatic hydrogen spin system were parsed in the Spinach package [33], a modern NMR simulation package running in the MATLAB environment, along with the corresponding values for chemical shifts and *J*-couplings retrieved from the Biological Magnetic Resonance Bank [30]. The purpose of our selection was a quantitative comparison regarding the potential lifetimes of adapted spin states among a series of molecules studied at the same level of theory. The rotational correlation time, $\tau_{c}$, was derived from experimental *T*_1_ measurements performed on Gly (50 mM in 100 % D_2_O solution at a temperature T = 304 K) using the following equation $T_{1}=\frac{3}{2}b_{IS}^{2}\tau_{c}$, where $b_{IS}=-\frac{\mu_{0}}{4\pi }\;\frac{\gamma^{2}\hbar }{r^{-3}}$ is the dipolar coupling constant. The latter parameter $b_{IS}$ was computed with SpinDynamica [34] using an interatomic distance between the two protons, r = 1.77 Å, derived from Gaussian09 simulations. The calculated value $\tau_{c}\approx 50\;ps$ was used for all simulations given that the molecular masses of the selected amino acids did not vary considerably. The differences between *T*_LLS_ at different correlation times were expected to be smaller than other factors such as interactions with the solvent, the removal of paramagnetic oxygen, and other spin interactions not considered in this paper.

For the calculation of complete Liouvillian, we employed the Bloch–Redfield–Wangsness relaxation theory [35] with full spherical-tensor based formalism, a modern approach. Only the aliphatic ^1^H nuclei were considered as the other protons bonded to nitrogen or oxygen were in fast exchange with the deuterated solvent. Other parameters can be checked in the attached notebook from the Supplementary Materials Figure S1 and Table S1.

In order to discover new long-lived spin states and compare computed values with existing experiments [19,20,36], one must simulate the Liouvillian of the considered spin system and compute its eigenstates [20]. Among these, there are a few that have a very small real part of the eigenvalue (the relaxation rate) and null imaginary part (oscillation frequency). For multiple-spin systems, these states may feature complex structures in terms of angular momenta operators and become impractical to consider theoretically as an intuitive pulse sequence to reach these complicated states via spin dynamics would be difficult to design. An alternative way to discover slowly relaxing states of the Liouvillian would be to thoughtfully design nuclear states that should be resilient to certain (selected) coherent evolution and relaxation mechanisms. One can generate long-lived states for ensembles of three spins or more with a specific combination of spin operators in order to inhibit specific relaxation processes at the same time as scalar evolution under the *J*-couplings network is warded off. As an example of slowly-relaxing states in 3-spin (*I*,*S*,*R*) ensembles, bilinear combinations of 3 *J*-coupled spins [20] were optimized in order to achieve the longest lifetime. The specific expression for these states is:

(1) $$Q^{ISR}=\;\frac{1}{\sqrt{3}}\left(\lambda_{IS}\vec{I}\cdot \vec{S}\;+\lambda_{SR}\vec{S}\cdot \vec{R}\;+\lambda_{IR}\vec{I}\cdot \vec{R}\;\right)$$

For these states, there is a specific choice of coefficients $\lambda_{ij}$ (i,j=I,S,R) that maximizes the time needed for the system to go back to equilibrium. We performed such calculations in order to provide optimal magnetization storage in long-lived states.

## 4. Conclusions

This study discusses several endogenous molecules in terms of their potential to support magnetization lifetimes in long-lived states, thus becoming candidates for biomarkers either for metabolomics or *d*-DNP-enhanced molecular imaging. The computational method for selecting adapted networks of protons is embedded in a new calculation approach for optimal LLS excitation and sustaining schemes in these molecules. The predicted magnetization lifetimes were in fair agreement with experimental data. Using proton spin magnetization exclusively, we propose biomarkers that circumvent expensive labeling with nitrogen-15 or carbon-13 isotopes, and, moreover, forego any modifications in the structures of molecules to host external markers.

## Figures and Tables

**Table 1:** *T*_1_ and *T*_LLS_ for the selected amino acids. The *T*_LLS_ values were also computed for the case of low magnetic field (*B*_0_ = 1 T). * Values reported from the available literature [16,19,20] (in some cases the experimental settings were different to ours).

Compound		Experimental		Theoretical	
	*T*_1_ of H^β^ (s)	*T*_LLS_ (s)	*T*_1_ H^β^ (s)	*T*_LLS_ (s)	*T*_LLS_ (s) at *B*_0_ = 1 T
Arg	0.9	-	0.81	3.38	3.22
Asp	1.6	7.48 *	0.99	7.64	13.15
Asn	1.3	10.7	0.99	8.333	15.45
Cys	1.1	13.1	0.99	9.69	22.88
Met	1.1	-	0.80	3.72	3.55
Phe	1.1	-	0.87	5.91	5.94
Ser	1.2	10.0	1.03	9.47	21.64
Thr	3.1	5.1	3.76	4.15	3.83
Val	2.1	-	1.44	3.1	3.09
Citrulline	-	-	0.83	1.86	1.65
Lactic acid	-	-	0.53	3.84	3.47
Citric acid	-	4.50 *	1.03	2.29	9.43
Fumarate	-	60 *	30.01	>100	>100
Methylfumarate	-	-	1.96	34.59	-
Dimethylfumarate	-	360 *	1.81	>100	-

**Table 2:** Predicted lifetime of *Q^ISR^* states for the chosen amino acids with *λ* coefficients of each spin product operator and the coupling constants between each pair of spins in a field of $B_{0}=1\;T$.

Compound	$T_{Q^{ISR}}$	$\lambda_{IS}$	$\lambda_{IR}$	$\lambda_{SR}$	$J_{IS}$	$J_{IR}$	$J_{SR}$
Asparagine	19.215	0.855	−0.100	0.098	−13.55	5.65	1.64
Aspartic Acid	19.305	0.854	−0.100	0.099	−13.41	5.51	1.55
Hystidine	20.833	0.852	−0.106	0.107	−13.68	5.78	1.43
Serine	19.723	0.852	−0.109	0.109	−10.52	4.81	1.32

## Supplementary Materials

The following are available online, Figure S1: The SLIC pulse sequence used to excite long-lived states in Asn, Cys and Ser.; Table S1: The parameters for SLIC pulse sequence used to excite long-lived states in Asn, Cys and Ser. Some of the theoretical aspects of the relaxation of long-lived spin order are presented in the supplementary information. Additionally, the Spinach notebook is given along with the Gaussian simulations and BMRB data used for simulation.
